# Development and content validation of the Healthcare Transition Outcomes Inventory for young adults with type 1 diabetes

**DOI:** 10.1186/s41687-019-0163-9

**Published:** 2019-12-19

**Authors:** Jessica Pierce, Karen Aroian, Elizabeth Schifano, Anthony Gannon, Tim Wysocki

**Affiliations:** 1 Nemours Children’s Hospital, 13535 Nemours Parkway, Orlando, FL 32827 USA; 2 University of Central Florida, 12201 Research Parkway, Orlando, FL 32826 USA; 3 Nemours Research Institute, 6900 Lake Nona Blvd, 5th Floor, Orlando, FL 32827 USA; 4 Nemours/Alfred I duPont Hospital for Children, 1600 Rockland Road, Wilmington, DE 19803 USA; 5 Nemours Children’s Specialty Care, 807 Children’s Way, Jacksonville, FL 32207 USA

**Keywords:** Young adults, Measure development, Type 1 diabetes, Healthcare transition

## Abstract

**Background:**

The literature on the specification and measurement of the outcomes of the healthcare transition from pediatric to adult centered-care is scarce and methodologically weak. To address these gaps, we conducted a series of studies to develop a multidimensional, multi-informant (young adults, parents, and healthcare providers) measure of healthcare transition outcomes for young adults with type 1 diabetes (T1D), the Healthcare Transition Outcomes Inventory (HCTOI). The current study describes the development and refinement of the HCTOI item pool.

**Methods:**

Following Patient Reported Outcomes Measurement Information System (PROMIS) standards, the research team conducted qualitative interviews to define six content domains of healthcare transition outcomes from the perspectives of multiple stakeholders, developed an initial item pool of the HCTOI based on the six domains, analyzed expert item ratings and feedback for content validation, and conducted cognitive interviews with informants (patients, parents, and healthcare providers) for further item pool refinement.

**Results:**

Qualitative findings revealed six healthcare transition outcome domains: 1) Biomedical markers of T1D control; 2) Navigation of a new health care system; 3) Possession of T1D self-management skills and knowledge; 4) Integration of T1D care into emerging adult roles; 5) Balance of parental involvement with autonomy; and 6) Attainment of T1D “ownership.” An initial pool of 88 items focused on the extent to which a young adult with T1D is successful on each of the six domains. Experts rated all content domains and all but six items as relevant. In addition to suggesting additional items, experts were concerned about the length of the measure, response burden, and whether every informant type would have sufficient knowledge to rate items in particular content domains. Cognitive interviews resulted in retaining all six content domains, but dropping some items and yielded fewer items for the healthcare provider version (47 items versus 54 items for the young adult- and parent-versions).

**Conclusions:**

Expert review and cognitive interviews confirmed that all six domains of HCT outcomes were relevant and both procedures resulted in retaining a sufficient number of clear and representative items for each content domain. The HCTOI represents the first multi-informant, rigorously developed item pool that comprehensively measures the multiple components of the transition from pediatric to adult specialty healthcare.

## Background

The prevalence of type 1 diabetes (T1D) in youth is increasing [5] and transitions from pediatric to adult care will increase accordingly. This healthcare transition (HCT) to adult care offers many challenges since young adults with T1D must balance the complex demands of T1D self-care with the more typical, but demanding, social and emotional changes experienced by this age group (e.g., work/college, social relationships, housing) [1, 16]. The outcomes of the HCT are therefore multidimensional, but existing measurement of HCT tends to be overly focused on biomedical outcomes (i.e., hemoglobin A1c) and neglect other important HCT dimensions (e.g., healthcare system navigation, disease management). Additionally, assessments of the various domains of HCT outcomes vary among studies [4, 9] and there is inconsistency about which outcomes to measure and the criteria indicating a successful HCT. This heterogeneity of HCT outcomes measures limits the ability to draw generalizable conclusions about the impact of the HCT on health and well-being and the efficacy of interventions focused on improving the HCT process. Such challenges are exacerbated by a lack of valid measures of transition outcomes to assess the multidimensional nature of HCT outcomes [11, 13].

Since the challenges of the HCT are multisystemic, requiring continuous interaction between patients, families, health care providers, and the medical system [15], these multiple outcome dimensions should also be considered from the perspectives of multiple stakeholders. Thus, a multidimensional, multi-informant HCT outcomes profile that yields pertinent data from young adults with T1D, parents, and healthcare providers would be a valuable contribution to clinical practice and research. We proposed to develop the Healthcare Transition Outcomes Inventory (HCTOI), a measure of HCT outcomes with versions to be completed by multiple informants, including young adults with T1D, parents, and healthcare providers. Such a measure would be useful in future studies that evaluate the impact of the HCT on YA health and well-being and for guiding the development and evaluation of interventions aimed to improve the HCT process.

To develop the HCTOI, we are following the rigorous standards embedded in NIH’s Patient Reported Outcomes Measurement Information System (PROMIS [3, 6]). Few measurement development and evaluation studies utilize such rigorous methods, but doing so for this work is essential given the complexity of the HCT process and the lack of valid HCT outcomes measures [11]. Development of a PROMIS item bank starts with a set of qualitative methods to produce a theoretically informed item pool. Iterations of the item pools are continuously refined as a result of expert review, cognitive interviewing, and other mixed methods. The item pool is then administered to a large, representative sample and the data undergoes psychometric analyses, which lead to further modification of the item pool. At the end of this process, an item bank is formed, which is ready for population norming, clinical validation, and responsiveness testing.

In this study, we describe the multi-phase development and refinement of the HCTOI item pool. Consistent with PROMIS methods, the specific aims were to: 1) define the transition success construct from the perspectives of multiple stakeholders, 2) generate an initial item pool, 3) refine the item pool through content validation by content area experts, and 4) conduct cognitive interviews with informants (i.e., young adult patients, parents, and a providers) to determine if the items elicited the intended meanings and whether each informant type would be able to rate the items. Our goal is to contribute to the conceptualization of transition outcomes and describe methods for developing measures for this complex construct.

## Methods

**Fig. 1:**
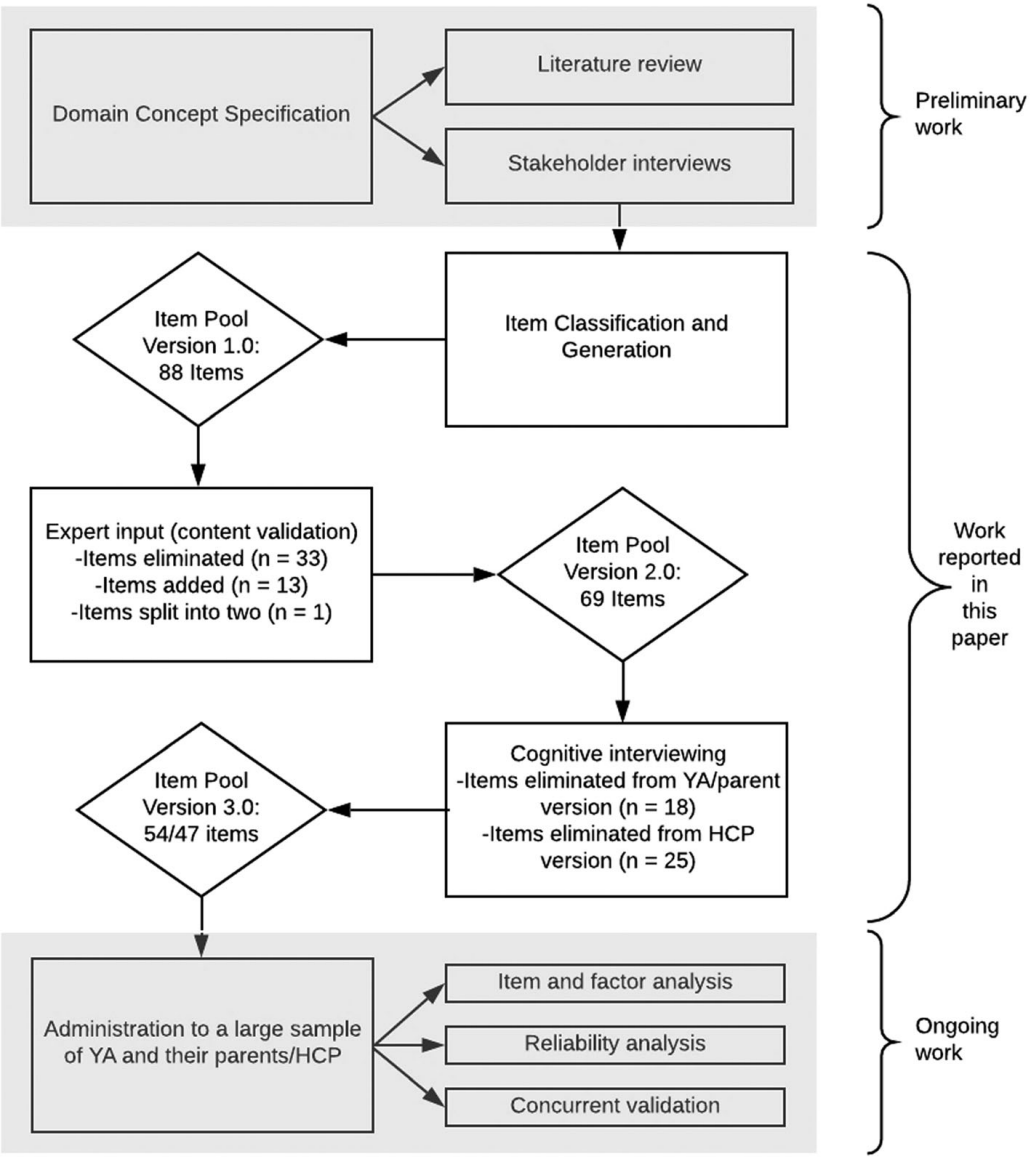
Process of developing the HCTOI item bank following PROMIS standards

Figure 1 outlines the iterative process for developing the HCTOI item pool using PROMIS methods. The institutional review board of the study site approved all study procedures, including an appropriate informed consent process for each stakeholder/informant group’s involvement in each element of the research.

### Defining the transition success construct

Our first step was to specify the transition success construct by obtaining perspectives of HCT stakeholders to define the objectives that an optimal HCT process should achieve. The specific methods of construct specification are described in detail elsewhere [10, 11] and are summarized here. Participants were 46 persons from five stakeholder groups who participated in semi-structured qualitative interviews: YA with T1D who recently transferred out of pediatric T1D specialty care; parents of YA with T1D; pediatric T1D specialty care providers; adult T1D specialty care providers; and healthcasre professionals or researchers with expertise in HCT for YA with T1D. Qualitative interview topics were based on an expansion of a validated model of transition readiness to include transition outcomes [10–12, 15] and included defining and describing successful HCTs, as well as cognitive, emotional, and behavioral indicators and exemplars of successful and unsuccessful HCTs.

### Initial item pool generation

Using the qualitative findings from our preliminary study [10], we developed an item pool for the HCTOI. While thoroughly reviewing the coded transcripts, author JP drafted items representing each of the six outcomes domains. Authors TW and KA, who were also intimately familiar with the coded transcripts, reviewed these initial items and made suggestions on item wording and additional items. We also determined the response format (i.e., Likert scale), scoring method, and instructions. During the item development phase, the item pool was generically worded to be completed by any informant type (e.g., “Keeps scheduled appointments with adult diabetes provider”).

### Content expert input

To further validate the content and refine the pool of items, the researchers engaged healthcare professionals who were involved with specialized HCT clinics designed to facilitate the HCT process for adolescents and young adults with T1D (i.e., transition clinics) and/or researchers with multiple publications and/or grant funding in HCT in T1D to review the items and provide feedback on the content, response format, and instructions. Experts were recruited through an invitational email sent by author JP. Experts were 10 healthcare professionals (2 combined pediatric/adult endocrinologists, 2 pediatric endocrinologists, 1 adult endocrinologist, and 5 pediatric health psychologists). Experts reported 6–14 years of research and/or clinical experience with T1D and 70% described their race/ethnicity as Non-Hispanic Caucasian.

Experts rated items for representativeness of the content domain on a 4-point scale, where 1 = *not representative* and 4 = *representative*. Only items that earned representativeness ratings > 3 from at least 8 of the 10 experts were retained in the item pool [7, 8]. Experts also rated items as *clear and concise* (i.e., extent to which each item was well written, distinct, and at an appropriate reading level) or *not clear and concise* [7, 8]. Experts provided suggestions for improvement of items they rated as a 1 or 2 for representativeness or as not clear/concise. They were also asked to suggest items that were missing from each domain, items that could be combined or eliminated, and any additional revisions/comments regarding each item and domain. Authors JP, TW, and KA systemically reviewed content validity ratings, and used comments from experts to guide revising and reducing the initial item pool.

### Cognitive interviewing

Since the HCTOI was developed as a multi-informant measure, all three informant types—young adults, parents, and adult diabetes care providers, participated in cognitive interviews on the item pool. To identify potential patients and parents, we conducted a query of the electronic health record of a pediatric health system serving patients in the Delaware Valley, North Florida, and Central Florida. The electronic health record query included contact information of patients with a diagnosis of T1D who were between ages 18 and 25 inclusive and did not have an outpatient encounter with T1D as the primary diagnosis in the previous year, as well as for their parent/caregiver. To facilitate purpopseful sampling, patients and parents on the query list were stratified into groups based on age, race/ethnicity, and duration of T1D diagnosis. A study coordinator randomly selected patient and parent participants from the stratified query lists and contacted them via phone to explain the study, confirm eligibility criteria, obtain verbal informed consent, and schedule the cognitive interview. Adult healthcare providers were recruited by author AG who sent invitational emails to adult endocrinologists in his professional network. The research team contacted interested providers to obtain verbal informed consent and schedule the cognitive interview.

The HCTOI items were divided into two sets of items with an approximately equal number of items per domain on each version in order to reduce informant burden. This resulted in two versions of the cognitive interview for patients and two parallel versions for parents and providers. Thus, every item was tested with four patients, three parents, and three providers. Interviews were completed over the phone and audio-recorded by one of three interviewers, the PI or one of the two research coordinators. All of the interviewers had experience conducting qualitative interviews. The primary cognitive interview technique was thinking aloud, which encourages participants to vocalize their thought processes as they responded to items [17]. Participants were instructed to read and respond to the item and to simultaneously say what was going through their mind, what they think the question was asking, and why they chose their response. Interviewers utilized verbal probes if they did not obtain adequate information through thinking aloud [17]. Probes included *“Can you repeat the question in your own words?*” and “*How did you arrive at your answer*?” Based on feedback received from expert review, we were interested in learning about whether providers had enough knowledge about their patients’ everyday lives to adequately respond to questions pertaining to certain domains (see Results section). Thus for providers, we also included the standard probe, “*Do you feel equipped to answer this question*?”

All participants completed a demographic information form prior to completing their interview. After reviewing instructions on thinking aloud, participants were instructed to proceed to practice thinking aloud using the following instructions recommended by Willis [17]: “*Try to visualize the place where you live and think about how many windows there are in that place. As you count up the windows, tell me what you are seeing and thinking about*.” Participants who had difficulty thinking aloud were prompted and encouraged to share their thoughts, no matter how unimportant they thought they were. After practicing, participants were instructed to proceed to the HCTOI items and response choices. Each item was shown on a separate page and participants had to click “Next” to proceed to the subsequent item. The entire interview was audio-recorded. The interviewer followed along with a script.

Following each interview, the interviewer listened to the full transcript and determined whether the item should be “kept as is,” “kept but revised,” or “dropped” based on the participant’s feedback. For all items marked as “keep but revise” and “drop,” the interviewer transcribed participants’ feedback verbatim. After all interviews were completed, JP, TW, and KA systemically reviewed the ratings and transcriptions. If the item was rated as “keep as is” across all interviews (i.e., four patients, three parents, and three providers), no changes were made to the item. If the item received at least one “keep but revise” and/or “drop,” we used the feedback obtained from interviewees to further guide revising and reducing the item pool.

## Results

### Defining the transition success construct

Rigorous content analysis of the interview data revealed six themes about indicators of a successful HCT. *Biomedical markers of T1D control,* which pertains to maintaining an in-range, or decreasing an elevated, HbA_1C_; low frequency of severe hypoglycemic events, diabetic ketoacidosis episodes, and early diabetes-related complications; and avoiding T1D-related hospitalizations. *Navigation of a new health care system* or establishing and maintaining continuity of healthcare post-discharge from pediatric T1D specialty care, including anticipating and overcoming healthcare system barriers and forming a collaborative, satisfying relationship with an adult T1D specialist. *Possession of T1D self-management skills and knowledge* for daily self-management of T1D, including executive functioning skills, such as forethought and planning. *Integration of T1D care into emerging adult roles*, such as school, work, socializing, and engaging in an adult lifestyle. *Balance of parental involvement with autonomy* whereby young adults find a balance that fosters adequate self-management with relying on parents when needed. *Attainment of T1D “ownership”* which refers to a frame of mind about T1D that accepts it as a chronic disease warranting prioritizing and taking responsibility for self-management. These findings are reported in a previous publication [10].

### Initial item pool generation

Through examination of coded transcripts, we drafted 88 items representing each of the six outcomes domains: Biomedical markers of T1D control (9 items); Navigation of a new healthcare system (16 items); Possession of T1D self-management knowledge and skills (24 items); Integration of T1D care into emerging adult roles (16 items); Balance of autonomy and parental support (13 items); and Ownership of T1D (10 items). Items were written using language frequently used by participants in the qualitative interviews (e.g., “takes charge of finding a new adult provider,” “makes diabetes management a priority,” and “fully accepts that diabetes management is a challenge”). A large number of items were generated because we strove to represent every example given by participants in the qualitative study and so that there would be a sufficient pool for future item reduction.

The initial response set was a 5-point Likert-scale (“Never true about me/my child/my patient” to “Always true about me/my child/my patient”) for most items except items in the Biomedical domain which used a fill-in-the-blank response format. The Biomedical domain items did not fit the Likert scale format because they described quantities rather than frequencies (e.g., most recent HbA_1C_, number of emergency department visits, and number of hospitalizations). The research team also developed the instructions for the HCTOI and time frame: “Below are a series of questions about the care of young adults with type 1 diabetes after they stop seeing a pediatric diabetes healthcare professional. Please rate each item for how they typically describe you and your behaviors in the last six months.” This time frame was chosen because it is recommended that patients with T1D see their diabetes healthcare provider every 3 to 6 months [2].

### Content expert input

Content validity ratings from the 10 experts who provided feedback revealed that 93%, or 82 of the 88 HCTOI items met the 80% agreement criterion for representativeness. The six items that did not meet the 80% threshold for agreement were removed. These included one item from the Biomedical markers domain in which experts noted that the item was not necessary; two items from the Navigation domain in which experts noted that items were too broad or vague; two items from the T1D knowledge and skills domain in which experts noted that items were too broad or unclear; and one item from the Integration domain in which experts noted that the item was not necessarily a determinant of a successful or unsuccessful transition. The mean representativeness rating of the remaining 82 HCTOI items was 3.68 (range = 3.61–3.79 across the six domains of transition success) on a scale of 1 to 4.

The research team also reviewed the comments from the HCT experts and further modified and reduced the item pool. Expert consensus revealed that the HCTOI was too long and impractical for administration. Thus, an additional 27 items were deleted based on suggestions from experts that they were redundant and/or not critical for measuring HCT success. For example, experts reported that young adults and parents may not be able to accurately assess whether the young adult possessed certain requisite T1D self-management skills and knowledge and even if the assessments were accurate, knowing” how to do something does not necessarily imply that it is being done. Thus, items assessing knowledge of specific T1D tasks were dropped (e.g., “Knows how to adjust insulin doses according to blood glucose level”), but items indicating behaviors related to those T1D tasks (e.g., “Adjusts insulin doses according to blood glucose level”) were kept.

Another 27 items received slight changes in wording, but their meaning was retained. For example, “Overcomes difficulties or barriers to health insurance” was changed to “Manages problems or potential problems with health insurance” and “Maintains acceptable quality of life while also managing diabetes carefully” was changed to “Maintains a balance between quality of life and managing diabetes.” The item “Has a satisfying relationship with adult diabetes provider” was divided into two items: “Is open and honest with adult diabetes care provider” and “Feels understood by adult diabetes care provider.”

Ten items were modified due to being too specific or not specific enough. For example, “Feels embarrassed or ashamed about diabetes” was changed to “Feels comfortable disclosing diabetes to others (for example: friends, romantic partners, coworkers, bosses, etc.” due to the feedback that the original item was too vague. The item “Has gaps of more than 6 months between visits with adult diabetes provider due to patient cancellations or no shows was changed to “Attended an appointment with adult diabetes provider” due to the feedback that the original item was too specific (i.e., the reason why there is a gap in care does not matter in determining a successful HCT).

Finally, 13 items were added based on suggestions of important topics that were not covered. Important topics included managing the costs associated with T1D care, driving when blood glucose levels are in a safe range, reviewing data patterns, getting to appointments on time, and making T1D management a routine part of life.

Several experts also commented that many parents and/or healthcare providers might not know the level of detail about the young adult in question in order to respond accurately to certain items (e.g., “Teaches friends and significant others about diabetes management”). However, the research team decided to retain those items in order to obtain additional feedback during cognitive interviews. Since most concerns were about whether the parents or healthcare providers would posses the requisite knowledge to rate certain items, “Unable to rate this item” was added to the response set on the parent- and healthcare provider-versions of the HCTOI. Thus, after dropping 33 items, modifying 37 items, and adding 14 items, the HCTOI contained 69 items at this point and subjected to cognitive interviewing with patients, parents, and providers.

### Cognitive interviewing

Patient participants for cognitive interviewing were eight young adults with T1D who were previously followed at the recruiting pediatric healthcare institution (19–24 years old; 11.6 years diagnosed with T1D; five males and three females; three Non-Hispanic Caucasian, four African American, and one Hispanic Caucasian). Parent participants in the sample were six parents of young adults with T1D who were previously followed at the recruiting pediatric healthcare institution (40–57 years old; five mothers, one father; 33.3% with bachelor’s or professional degree). Provider participants in the sample were six adult endocrinologists (7–28 years of experience treating patients with T1D; 66.7% Non-Hispanic Caucasian). Although both pediatric and adult providers participated in the qualitative interviews about HCT outcomes that generated content for the HCTOI, only adult providers were included in cognitive interviews because they represent the provider informants who will be using the measure to evaluate the outcomes of the HCT.

For patients and parents, 20 items were rated as “keep as is” and for providers, 14 items were rated as “keep as is.” No changes were made to those items. The remaining 49 patient and parent items and 55 provider items were considered for deletion or modification. Through discussion and consensus by the research team, incorporating results from the interviews, 15 patient and parent items were removed and 22 healthcare provider items were removed. Similar to the feedback from expert review, adult endocrinologists suggested that they would be unable to rate many of the Parental Support/Autonomy and Integration of T1D items. Although they did not negate the relevance of this content domain as important HCT outcomes, they suggested that practice demands and time constraints interfered with their ability to gather this information from their patients. Other items were removed because during the thinking aloud procedure and probing, it was determined that they were not applicable to all young adults (e.g., “argues with parent(s) about the costs of diabetes care.”), were too specific (e.g., “gets to scheduled diabetes-related appointments on time”), were too vague (e.g., “good results from managing diabetes gives feeling of accomplishment”) or were not important for assessing HCT outcomes (“is satisfied with most recent A1C result”). We also added two items “I have consistent routines for monitoring blood glucse levels and taking insulin” and “I know how to find a new diabetes care provider if one were needed” based on suggestions by interviewees that these were important topics to address.

We modified 26 of the items based on feedback that the item was difficult to understand or ambiguous, or was misinterpreted by participants. Some revisions reflected verbatim comments made by participants while they were thinking aloud and explaining what they thought the item meant. For example “copes with the burden associated with managing diabetes” was changed to “has good strategies for coping when diabetes gets stressful.” Several additional examples were also added to items to clarify their meaning. For example, several participants did not understand the term “recreational drugs,” so examples, such as marijuana, cocaine, and “pills not prescribed for me” were added for clarification. Based on suggestions that some young adults only see their adult endocrinologist every 6 months, the time frame in the instructions was changed from “in the past 6 months” to “in the past year.” The items “drives only when blood glucose is in a safe ranges” and “stops driving at the earliest signs of low blood glucose” were combined into a single item, as were “thinks about diabetes when making decisions about whether to drink alcohol” and “thinks about diabetes when making decisions about whether to use recreational drugs.” Thus, after dropping 17 young adult/parent items and 24 healthcare provider items, modifying 26 items, and adding 2 items, the HCTOI contained 54 items for the young adult and parent versions of the HCTOI and 47 items for the healthcare provider version ready to be tested in a large validation study.

The response style and items for the Biomedical domain was also changed based on feedback from interview participants. For example, the majority of participants suggested that it would be clearer to report the number of hypoglycemic events, diabetes-related hospitalizations, and diabetes-related emergency department visits as a quantity rather than as whether or not they occurred within the specified time range (e.g., “Number of severe low blood sugar episodes (seizure, loss of consciousness, or required glucagon) you’ve had” instead of “Has had a severe low blood sugar episode [i.e., in the past six months]”).

## Discussion

The results of the current study support the content validity of the HCTOI item pool, which will be further tested and refined before dissemination. Consistent with PROMIS methods, we began with a stakeholder-driven, hypothesis-generating study aimed to define the six domains of a successful HCT and utilized this data to develop an initial item pool. We then subjected these initial items to expert review and cognitive interviewing of young adults, parents and adult care providers, two additional steps following PROMIS methods, which resulted in an item pool ready to be tested in a large validation sample. Development of the HCTOI incorporated feedback from multiple stakeholders and experts, an iterative and purposeful process to allow continuous refinement while also optimizing its face and content validity among informant groups. Defining the HCT success construct and content validating items for measuring HCT success using rigorous PROMIS methods is a first and important step for comprehensively assessing and tracking the outcomes of the healthcare transition for young adults with T1D from the perspectives of multiple stakeholders. A measure assessing the multidimensional nature of HCT outcomes, permits the advancement of HCT research which has traditionally relied on biomedical outcomes and neglected the social-ecological nature of the HCT process [11].

Much of the exisiting literature on HCT focuses on preparing adolescents and YA for a successful HCT (i.e., transition readiness), but less emphasis has been placed on how to measure whether the HCT was actually successful. This study described the development of such a measure, the HCTOI, for YA with T1D. The HCTOI will be instrumental in determining whether YA who demonstrate HCT readiness in pediatric care are actually successful in adult T1D care, providing a means to evaluate longitudinal relations between HCT readiness and outcomes and trajectories of HCT outcomes over time [11]. Although we would expect HCT readiness to predict successful HCT outcomes (i.e., better scores on the HCTOI), it is likely that some YA with high levels of HCT readiness would have poor HCT outcomes and some YA with low levels of HCT readiness would have more favorable HCT outcomes. Identification of different HCT trajectories and linking these trajectories to other clinical outcomes would permit identification of subgroups of young adults who might benefit from various components of HCT interventions. Once validated, the HCTOI will also all for comparison of results across future HCT intervention trials [11].

The next phase of HCTOI development, currently in progress, is a large cross-sectional validation study. We seek to enroll 200 young adults with T1D along with their parents and adult healthcare providers to complete the HCTOI and a battery of related measures to examine the psychometric properties of the HCTOI. We will conduct confirmatory factor analysis to examine the hypothesis that the HCTOI will yield six reliable factors consistent with the six domains of successful HCT outcomes identified in our qualitative work. We will also evaluate internal consistency, inter-rater agreement, construct validity (associations with other conceptually related, validated measures) and criterion validity (associations with measures of T1D adherence, glycemic control, and other biomedical outcomes). Item analyses will include examination of item distributions, item-total correlations, and inter-item correlations. Careful refinement of items is expected to result in a briefer, practical and acceptable measure that is ready for clinical dissemination and research use.

The present study has several limitations. The HCTOI was designed specifically for young adults with T1D, so it is possible that a disease-specific measure may not be readily generalizable for other medical conditions. However, the PROMIS methods used in the present study can inform the adaptation of HCTOI items for other populations [14]. For example, Schwatz and colleagues (2017) used similar PROMIS methods to develop a measure of transition readiness for adolescents and young adults with cancer [14]. Although our sample size for the cognitive interviews does meet the criterion for recommended number of interviews, we were unable to conduct additional rounds of interviews (Beatty & Willis 2007). This limitation may be offset somewhat by the prior stakeholder engagement steps taken to develop the HCTOI (i.e., qualitative interviews, expert review). Additionally, the only providers who participated in cognitive interviews were adult endocrinologists, whereas other provider types (e.g., nurse practitioners, generalists) may also provide adult diabetes care. When the healthcare setting utilizes a multidisciplinary approach, additional provider types are even more likely. Thus, revisions to the HCTOI based on provider input may have limited generalizability. Finally, the rigorous PROMIS process for measure development and validation is time-consuming and labor-intensive, and additional steps are required before the development and validation of the HCTOI will be complete.

## Conclusions

The HCTOI is being developed to provide a comprehensive assessment of HCT outcomes through its multidimensional, multiple stakeholder approach. Together with validated measures of HCT readiness, the HCTOI can be used to identify trajectories of HCT readiness and success to identify who will benefit from interventions. The rigorous PROMIS methods used are essential given the complexity of the HCT process and the lack of valid HCT outcomes measures. The content validation and cognitive interviewing steps conducted as part of HCTOI development confirms our preliminary work indicating that HCT outcomes are multidimensional and require multiple stakeholder perspectives. Healthcare providers working with YA with T1D should recognize that evaluation of HCT outcomes goes above and beyond the YA’s HbA1c level and requires multiple components and perspectives. Target risks and barriers toward achieving the multiple domains of successful HCT outcomes will be crucial in the development of future HCT interventions.

## Data Availability

Please contact author for data requests.

## Abbreviations

HCT: Healthcare transition
HCTOI: Healthcare Transition Outcomes Inventory
PROMIS: Patient Reported Outcomes Measurement Information System
T1D: Type 1 diabetes
YA: Young adult
